# Effect of Monomer Sequence along Network Chains on Thermoresponsive Properties of Polymer Gels

**DOI:** 10.3390/gels4010022

**Published:** 2018-03-10

**Authors:** Shohei Ida, Toru Kawahara, Hidekazu Kawabata, Tatsuya Ishikawa, Yoshitsugu Hirokawa

**Affiliations:** Department of Materials Science, The University of Shiga Prefecture, 2500 Hassaka, Hikone, Shiga 522-8533, Japan; uspmatpolyst1@gmail.com (T.K.); uspmatpolyst2@gmail.com (H.K.); uspmatpolyst3@gmail.com (T.I.)

**Keywords:** gel, thermoresponsive property, monomer sequence, co-crosslinking, copolymerization, acrylamide derivative, swelling, volume phase transition

## Abstract

The effect of monomer sequence along the network chain on the swelling behavior of polymer gels should be clarified for the advanced control of swelling properties of gel materials. To this end, we systematically investigated the swelling properties of poly(acrylamide derivative) gels with the same composition but different monomer sequence by utilizing two gel synthetic methods: copolymerization giving a random network and co-crosslinking giving a blocky network. Both of the copolymerization and the co-crosslinking gels were prepared from the combination of two of the three following monomers: hydrophilic *N*,*N*-dimethylacrylamide (DMAAm), hydrophobic *N*-*n*-butylacrylamide (NBAAm), and thermoresponsive *N*-isopropylacrylamide (NIPAAm) with various monomer compositions. The swelling measurement of the obtained gels showed totally different behaviors between the copolymerization and the co-crosslinking gels, even with the same monomer composition. The copolymerization gels had the average property from the two monomers, depending on monomer composition, because random monomer distribution changed the affinity of each network chain to water. On the other hand, the co-crosslinking gels behaved as if two components independently contributed to the swelling properties, probably due to the domain structure derived from two kinds of prepolymers.

## 1. Introduction

Since the discovery of volume phase transition of polymer gels by Tanaka et al. [1,2,3], research on stimuli-responsive gels has been actively conducted. The most representative example is poly(*N*-isopropylacrylamide) (PNIPAAm) gel, which shows phase transition at around 33 °C in pure water and is anticipated to be useful in various applications [3,4,5,6,7,8,9]. In order to develop highly functional thermoresponsive materials, it is important to precisely control the thermoresponsive properties, including transition temperature, range of volume change, and transition speed, according to the purpose. The thermoresponsive property in water originates from the balance of hydrophilicity and hydrophobicity in the network chains: the former dominates the hydration at low temperature and the latter affects the aggregation of hydrophobic groups at high temperature. Therefore, an appropriate design of hydrophilicity/hydrophobicity balance in the structure is required for the control of thermoresponsive properties.

A general approach to control the swelling properties of polymer gels is the combination of plural monomers by copolymerization. Copolymerization of a monomer which gives a thermoresponsive gel such as *N*-isopropylacrylamide (NIPAAm) with another monomer easily creates a shift of the transition temperature due to changing the balance of hydrophilicity and hydrophobicity of network chains by feed ratio of the monomers [10,11,12,13,14,15]. Moreover, we have recently found that the adequate combination of hydrophilic monomer and hydrophobic monomer, both of which could not give a thermoresponsive polymer alone, could produce thermoresponsive gels showing sharp transition [16]. Thus, copolymerization of two kinds of monomers is a versatile method to control the thermoresponsive properties by regulating the balance of hydrophilicity and hydrophobicity.

**Figure 1:**
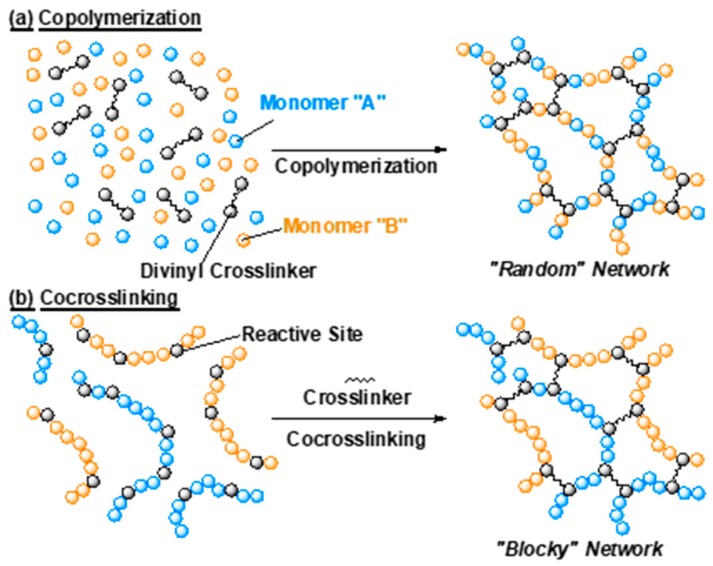
Schematic illustrations of gel network structure consisting of two kinds of monomer prepared by (**a**) copolymerization and (**b**) co-crosslinking.

Importantly, when combining two types of monomers, the gel properties should be strongly affected by the monomer sequence in the network chains, as the properties of linear polymers are greatly different between random sequence and block sequence. This indicates that a detailed understanding of the effect of monomer sequence is required for the advanced control of the swelling properties of the gels. However, commonly used radical copolymerization often produces only random sequences of two monomers in the network as shown in Figure 1a.

On the other hand, it is known that a so-called amphiphilic conetwork (APCN) exhibits a characteristic swelling behavior. APCN has a structure combining two kinds of polymers, and shows the swelling behavior reflecting the properties of the two polymers [17,18]. For example, the combination of hydrophilic and hydrophobic polymers gives an APCN which can swell both in water and organic solvents [19,20,21,22,23,24]. Thermoresponsive polymers can be used as a constituent polymer of APCN, and the combination of thermoresponsive polymers and other polymers affords remarkable effects to the gels, such as improvement of response speed and thermoresponsive toughening [25,26,27,28,29,30,31,32,33,34]. Such an APCN structure is regarded as a contrasting sequence to a copolymerization gel having a random sequence. Therefore, systematic analysis of APCN and copolymerization gels using the same monomer combination would lead to understanding of the swelling behavior of the gel composed of two types of monomers, and it should contribute to the design criteria for the swelling properties of polymer gel materials.

In this paper, we focus on the effect of monomer sequence along network chains on the swelling properties of poly(acrylamide derivative) gels. To this end, the gels with the same monomer composition but a different monomer sequence along network chains were prepared by utilizing two synthetic methods. One method was copolymerization of two kinds of monomers to afford the *random* network in which each polymer chain between crosslinking points is a random copolymer like “-ABBABAAB-” (A and B stand for monomer units) as shown in Figure 1a. For the other method, to prepare the APCN structure, we utilized post-polymerization crosslinking methods using activated-ester chemistry [35]. Two kinds of poly(acrylamide derivative)s were employed for the crosslinking, and this “co-crosslinking” produces a *blocky* network in which all the network chains between crosslinking points are homopolymer like “-AAAAAA-” or “-BBBBBB-” (Figure 1b). We prepared copolymerization and co-crosslinking gels in various combination using three kinds of monomers with different affinity to water, which were hydrophilic, hydrophobic, and thermoresponsive, respectively. The swelling behavior in water of these gels with the same composition but different sequence was systematically investigated to elucidate the effect of network chain sequence on the thermoresponsive properties of the gels. It revealed that the copolymerization gels showed the averaged properties of the employed monomers and produced a change in the response temperature, while the co-crosslinking gels behaved as if the two components independently functioned and afforded a change in the swelling degree while keeping a constant response temperature.

## 2. Results and Discussion

### 2.1. Gel Synthesis by Copolymerization and Co-crosslinking

**Figure 2:**
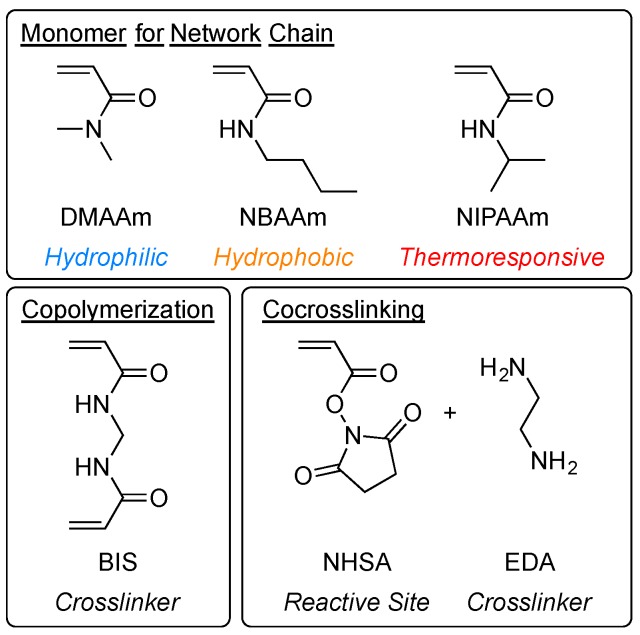
Chemical structure of monomers and crosslinkers for the synthesis of copolymerization and cocrosskinking gels. DMAAm = *N*,*N*-dimethylacrylamide; NBAAm = *N*-*n*-butylacrylamide (NBAAm); NIPAAm = *N*-isopropylacrylamide; BIS = *N*,*N’*-methylenebisacrylamide; NHSA = *N*-(acryloyloxy)succinimide; EDA = ethylenediamine.

In order to clarify the effect of monomer sequence along network chains on swelling behavior of gels, copolymerization and co-crosslinking gels were prepared in various combinations using three kinds of monomers, as shown in Figure 2. These monomers give polymers with different affinity to water; hydrophilic *N*,*N*-dimethylacrylamide (DMAAm), hydrophobic *N*-*n*-butylacrylamide (NBAAm), and thermoresponsive NIPAAm.

Copolymerization gels were synthesized via radical copolymerization of two kinds of the acrylamide derivatives in the presence of the divinyl crosslinker, *N*,*N’*-methylenebisacrylamide (BIS), with various monomer feed ratios. The copolymerization gels were supposed to possess a random monomer sequence in the network chains because the structural similarity of the monomers gives the same polymerization reactivity. The reaction was conducted in methanol as solvent due to cosolvency, and the reaction time was set for 24 h, which was supposed to be long enough for the completion of the polymerization. Therefore, we could regard the feed ratio as the composition of the copolymerization gels. After the completion of gelation, methanol in the gel was replaced by pure water by means of immersion of the gels into large amounts of water for several days.

Co-crosslinking gels were obtained by a crosslinking reaction of two kinds of prepolymers containing activated ester groups, which react efficiently with primary amines [36]. The co-crosslinking gels are supposed to have a blocky network sequence because of the synthetic procedure using two kinds of polymers. The prepolymers were prepared by radical copolymerization of each acrylamide derivative monomer with the monomer carrying activated ester, *N*-(acryloyloxy)succinimide (NHSA). The obtained polymers were characterized by size-exclusion chromatography (SEC) and ^1^H NMR analyses. The charactirization clarified that all the prepolymers had similar molecular weight and NHSA content as shown in Table 1. Then, the two kinds of prepolymers were dissolved in *N*,*N*-dimethylformamide (DMF), which is a good solvent for all the prepolymers, at various ratios to change the composition of the gels, and were then reacted with ethylenediamine (EDA) as crosslinker. After the completion of gelation, DMF in the gel was replaced by pure water in the same process used for the copolymerization gels. Herein, it should be noted that the copolymerization gels and the co-crosslinking gels were prepared at the same feed concentration of monomer unit and crosslinker, which gave almost the same crosslinking density under the assumption that all monomers and crosslinkers were completely consumed.

### 2.2. Hydrophilic/Hydrophobic Combination

**Figure 3:**
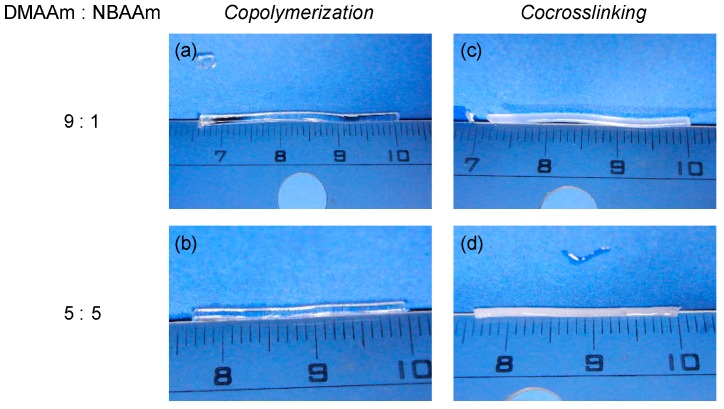
Appearance of (**a**,**b**) copolymerization and (**c**,**d**) co-crosslinking gels obtained from DMAAm and NBAAm with various monomer unit composition; DMAAm:NBAAm = (**a**,**c**) 9:1 and (**b**,**d**) 5:5.

The swelling properties of the copolymerization and the co-crosslinking gels with a variety of compositions were systematically compared. First, the combination of hydrophilic DMAAm and hydrophobic NBAAm was examined. The copolymerization gels and the co-crosslinking gels were different in their appearance as shown in Figure 3. The copolymerization gels were transparent in water at room temperature even with the NBAAm contents in the network chains reaching 50% (Figure 3b). On the contrary, the co-crosslinking gels showed turbidity during solvent replacement by immersion into water even with hydrophobic NBAAm composition as low as 10% (Figure 3c).

This difference in appearance was supposed to be derived from the change of the network structure. Focusing on the hydrophobic moieties, NBAAm monomer units randomly distributed in the network of the copolymerization gels, while PNBAAm blocks were introduced in the co-crosslinking gels due to the difference in the preparation method. Therefore, the hydrophobicity of NBAAm units in the co-crosslinking gels affected the swelling state in water much more strongly than did that of the copolymerization gels with the same composition. That is, the aggregation of hydrophobic blocks easily occurred in the co-crosslinking gels. When this aggregation reached the size of the wavelength of the visible light, the turbidity was observed. In the co-crosslinking gels, this aggregation occurred even with low NBAAm content.

**Figure 4:**
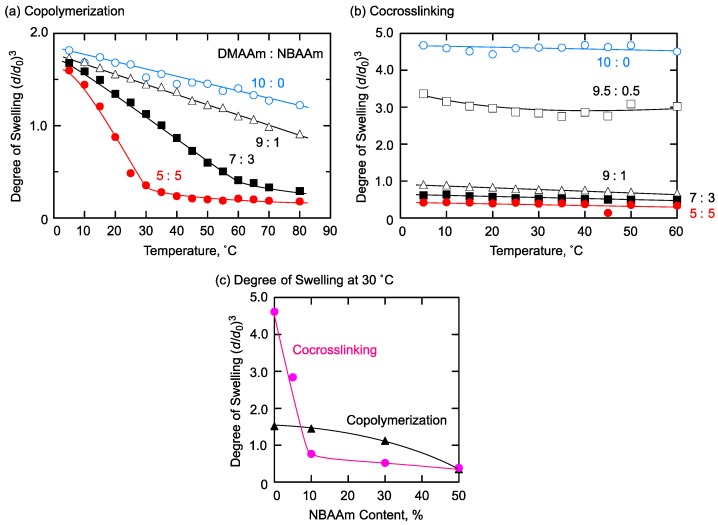
Swelling behavior of DMAAm/NBAAm (**a**) copolymerization and (**b**) co-crosslinking gels; (**c**) Relation between NBAAm content and the degree of swelling at 30 °C. Preparation condition of copolymerization gels: [DMAAm] + [NBAAm] = 1400 mM, [BIS] = 48.6 mM, [AIBN] = 55.6 mM in methanol at 55 °C. Preparation condition of co-crosslinking gels: [DMAAm unit] + [NBAAm unit] = 1400 mM, [NHSA unit] = [amino groups in EDA] in *N*,*N*-dimethylformamide (DMF) at room temperature.

Then, the swelling behavior against temperature change of the obtained gels was investigated. Here, the degree of swelling was determined as the ratio of the volume at each temperature against the initial volume at the preparation state. We have recently reported that the copolymerization gel of hydrophilic and hydrophobic acrylamide derivatives with appropriate monomer ratio showed thermoresponsive properties with a sharp volume change in water [16]. The combination of DMAAm and NBAAm was the typical example. Increasing NBAAm content of the copolymerization gel gave a large decrease in the swelling degree against temperature rising, as shown in Figure 4a. Particularly, the gels with DMAAm:NBAAm = 5:5 showed sharp shrinking.

In contrast, the co-crosslinking gels showed different behavior from that of the copolymerization gels as shown in Figure 4b. Introduction of NBAAm blocks to the co-crosslinking gels led to the decrease in the degree of swelling irrespective of the NBAAm content, and all composition of the gels did not give any change in the degree of swelling against temperature change. Here, there was a large difference between the degree of swelling of the copolymerization and that of the co-crosslinking gels with DMAAm:NBAAm = 10:0. This was probably due to the difference in the crosslinking efficiency in both methods. We employed the same monomer/crosslinker concentration at the two preparation systems, but the crosslinking efficiency was difficult to strictly control at this stage. Particularly, the solubility of the polymer to the reaction solvent was considered to affect the gelation behavior in the co-crosslinking reaction. As shown in the next section, such differences were not observed in the case of PNIPAAm gel. Thus, the solvent effect on the swelling degree appears in the case of PDMAAm co-crosslinking gel.

The difference in the swelling behavior of the copolymerization and the co-crosslinking gels was also clearly observed in the relation of NBAAm content and the degree of swelling at 30 °C as shown in Figure 4c. In the case of the copolymerization gels, the degree of swelling gradually decreased as the NBAAm content increased. On the other hand, the degree of swelling of the co-crosslinking gels sharply decreased at around 5~10% NBAAm content.

These results could be interpreted as the effect of the network chain sequence of the copolymerization and the co-crosslinking gels. In the copolymerization gels, hydrophilic and hydrophobic monomer units were randomly distributed along the network chains, and an increase in the content of NBAAm afforded alternating structures of DMAAm and NBAAm in the network chains [16]. This structure is similar to PNIPAAm gels in which the hydrophilic amide group and hydrophobic isopropyl group adjoined in one monomeric unit to realize an adequate balance of hydrophilicity and hydrophobicity for thermoresponsiveness. Similarly, the adequate balance of hydrophilicity/hydrophobicity was realized in hydrophilic/hydrophobic copolymerization gels to show thermoresponsive swelling behavior. On the other hand, since each monomer unit was separately introduced in the network in the case of the co-crosslinking gels, the swelling behavior was determined by the balance of hydrophilicity of PDMAAm and hydrophobicity of PNBAAm. Furthermore, this blocky sequence easily induced the aggregation of hydrophobic blocks in water to cause a decrease in the degree of swelling. The effect of hydrophobicity of PNBAAm became superior to the hydrophilic effect of PDMAAm at around 10% NBAAm content, and the sharp decrease of the degree of swelling was observed in this region. The blocky sequence also prevented the formation of amphiphilic local structures like copolymerization gels, and, therefore, the co-crosslinking gels did not show any thermoresponsiveness. Thus, the difference in network chain sequence strongly affected the swelling behavior of the gel prepared as a combination of hydrophilic and hydrophobic monomers.

### 2.3. Thermoresponsive/Hydrophobic Combination

**Figure 5:**
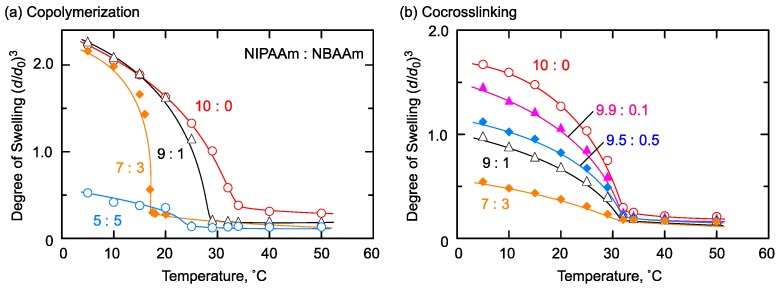
Swelling behavior of NIPAAm/NBAAm (**a**) copolymerization and (**b**) co-crosslinking gels. Preparation condition of copolymerization gels: [NIPAAm] + [NBAAm] = 1400 mM, [BIS] = 48.6 mM, [AIBN] = 55.6 mM in methanol at 55 °C. Preparation condition of co-crosslinking gels: [NIPAAm unit] + [NBAAm unit] = 1400 mM, [NHSA unit] = [amino groups in EDA] in DMF at room temperature.

The difference in the network chain sequence is supposed to strongly affect the thermoresponsive property of the gels, because the sequence relates to the hydration structure. Then, the effect of network chain sequence on the combination of thermoresponsive and hydrophobic monomers was examined by employing NIPAAm and NBAAm for copolymerization and co-crosslinking gel synthesis. Both synthetic methods produced gels showing shrinkage in response to temperature change in water, but increasing hydrophobic units had different effects on the swelling behavior, as shown in Figure 5.

As shown in Figure 5a, incorporation of hydrophobic NBAAm by copolymerization led to a lower shrinking temperature than that of PNIPAAm homopolymer gels until the NBAAm content reached 30%. This tendency was reasonable as it was previously observed in the case of thermoresponsive polymers obtained by copolymerization of NIPAAm with hydrophobic monomers [11]. The thermoresponsive property of PNIPAAm gel strongly relates to the balance of hydrophilicity and hydrophobicity. That is, hydration by amide groups leads to absorption and retention of water in the network at lower temperatures. Increasing temperature weakens this hydration effect, and aggregation by hydrophobic isopropyl groups induced volume collapse of the gels. In the NIPAAm/NBAAm copolymerization gel, randomly incorporated hydrophobic monomers suppressed the hydration of the network and strengthened hydrophobic interactions by butyl groups, resulting in a decreased shrinking temperature compared to that of the PNIPAAm gel. Notably, the copolymerization gel with NIPAAm:NBAAm = 5:5 seemed to show higher shrinking temperature than the gel with NIPAAm:NBAAm = 7:3. However, this gel was in the shrunken state even at 5 °C, and it showed turbidity and deformed at around 15 °C. After that, the macroscopic volume change of this gel may not follow the temperature change, and the apparent shrinking temperature would become higher.

On the other hand, the co-crosslinking gels showed the same transition temperature at around 32 °C regardless of the hydrophobic monomer composition, while the degree of swelling decreased with increasing NBAAm content as shown in Figure 5b. This suggests the presence of the domain structure in the network, which separately and independently comported. The co-crosslinking gels were prepared by a post-polymerization crosslinking reaction of two kinds of prepolymers. This preparation method produced a gel in which each network chain between the crosslinking points consisted of either PNIPAAm or PNBAAm homopolymer. This “blocky” sequence formed thermoresponsive and hydrophobic domains derived from each homopolymer chain. As indicated above, the thermoresponsive property of gels is derived from the affinity of network chains to water. Therefore, the same shrinking temperature was supposed to be due to the presence of PNIPAAm domains in NIPAAm/NBAAm co-crosslinking gels, because the affinity to water of thermoresponsive moieties in the co-crosslinking gels was equal to that of PNIPAAm homopolymer gels regardless of the presence of hydrophobic monomer units. At the same time, the hydrophobic domains in the co-crosslinking gels was attributed to the degree of swelling by aggregation in water.

Thus, the monomer sequence of network chains strongly affects the swelling properties of thermoresponsive gel’s incorporated hydrophobic moieties. Randomly distributed NBAAm units contributed to the decrease of transition temperature, while hydrophobic block structure affected the degree of swelling without change of the transition temperature.

### 2.4. Thermoresponsive/Hydrophiliic Combination

**Figure 6:**
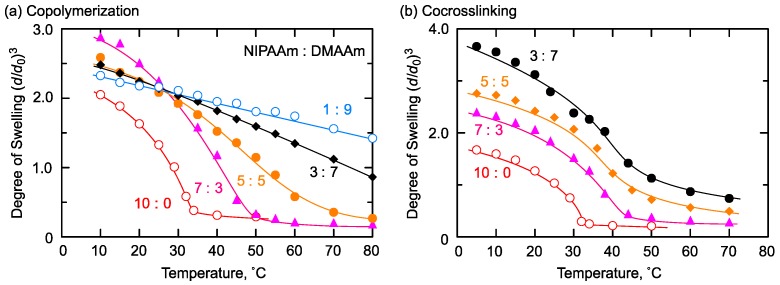
Swelling behavior of NIPAAm/DMAAm (**a**) copolymerization and (**b**) co-crosslinking gels. Preparation condition of copolymerization gels: [NIPAAm] + [DMAAm] = 1400 mM, [BIS] = 48.6 mM, [AIBN] = 55.6 mM in methanol at 55 °C. Preparation condition of co-crosslinking gels: [NIPAAm unit] + [DMAAm unit] = 1400 mM, [NHSA unit] = [amino groups in EDA] in DMF at room temperature.

A similar phenomenon to that of the thermoresponsive/hydrophobic combination was observed in the case of the thermoresponsive/hydrophilic combination. The copolymerization and the co-crosslinking gels from thermoresponsive NIPAAm and hydrophilic DMAAm were prepared, and the temperature dependence of the degree of swelling was measured in pure water (Figure 6). The copolymerization gels showed an increase in the shrinking temperature as the DMAAm content in the network increased (Figure 6a). On the other hand, the transition temperature of the co-crosslinking gels (NIPAAm:DMAAm = 7:3, 5:5, 3:7) was almost the same but higher than that of the PNIPAAm homopolymer gel (Figure 6b).

This difference of shrinking behavior could be also attributed to the difference in monomer sequences of the network chains, similar to that in NIPAAm/NBAAm gels. The randomly distributed DMAAm in the copolymerization gels strengthened hydrophilicity of the network, and led to the increase of transition temperature. On the other hand, DMAAm and NIPAAm units were incorporated in a blocky structure in the co-crosslinking gels, and the thermoresponsive domains could work independently. This led to almost the same transition temperature in the co-crosslinking gels. However, hydrophilic polymers affected the hydration structure of the gel, and the transition temperature was varied to be higher than that of the PNIPAAm homopolymer gel. Moreover, increasing hydrophilic moieties in the co-crosslinking gels simply produced an increase in the degree of swelling throughout the examined temperature.

**Figure 7:**
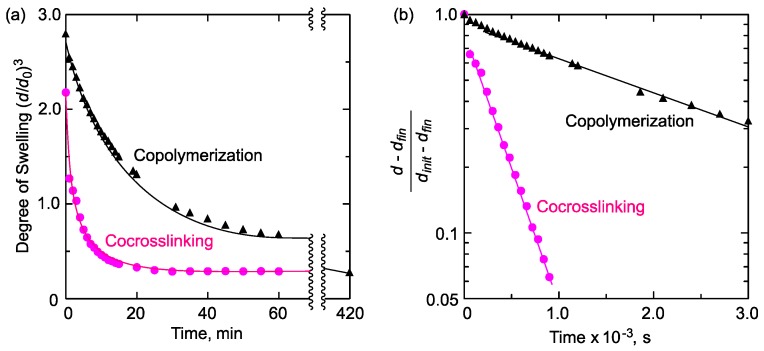
(**a**) Shrinking behavior of the copolymerization and the co-crosslinking gels of NIPAAm and DMAAm (7:3) against a temperature jump from 5 to 50 °C and (**b**) kinetic analysis of shrinking behavior upon temperature jump. When an isotropic shrinking is observed, the relaxation time (*τ*) can be determined from the slope of the line (−1/*τ*)

Furthermore, the sequence effects on the shrinking rate was evaluated. The copolymerization and the co-crosslinking gels with the composition of NIPAAm:DMAAm = 7:3 were immersed into cold water at 5 °C to reach the equilibrium swelling state. Then, the gels were quickly transferred to 50 °C hot water, and the time-dependent change of the degree of swelling was observed. As shown in Figure 7a, both gels smoothly shrunk just after the elevation of temperature without an induction period, but the co-crosslinking gel shrunk much more rapidly than did the copolymerization gel.

Next, we performed a quantitative analysis of the shrinking kinetics of the co-crosslinking and the copolymerization gels. For the swelling/shrinking of cylinder-shaped gels, the time dependence of diameter change can be described as

(1) $$\frac{d-d_{fin}}{d_{init}-d_{fin}}=\frac{6}{\pi^{2}}exp\left(-\frac{t}{\tau }\right)$$

where *d*_init_, *d*_fin_, and *τ,* respectively, stand for the equilibrium diameter at the initial swelling state, the equilibrium diameter at the shrunken state, and the relaxation time constant, when *t* is larger than *τ* and no phase separation occurs [37,38,39]. We applied this equation for the results of Figure 7a and determined *τ* for the co-crosslinking and the copolymerization gels: *τ* = 3.4 × 10^2^ s for the co-crosslinking gel and *τ* = 2.5 × 10^3^ s for the copolymerization gel (Figure 7b). For cylindrical gels, the relaxation time (*τ*) is related to the cooperative diffusion coefficient (*D*):

(2) $$D=\frac{3d_{fin}^{\;\;\;\;2}}{8\pi^{2}\tau }$$

From this relationship, we estimated *D* at 8.6 × 10^−7^ cm^2^·s^−1^ for the co-crosslinking gel and 1.1 × 10^−7^ cm^2^·s^−1^ for the copolymerization gel. These values indicated that the co-crosslinking gel shrunk much faster than did the copolymerization gel. Furthermore, it can be considered that the dehydrated water spilled out of the gels much more smoothly in the case of the co-crosslinking gel, since the value *D* is related to the modulus of polymer chains and the frictional coefficient between the network and water.

This quick shrinking of the co-crosslinking gel is due to the presence of hydrophilic domains derived from PDMAAm chains. It has been reported that incorporation of hydrophilic polymers or domains into the thermoresponsive gel accelerates the shrinking rate because dehydrated water can effectively spill out of the gels through the hydrophilic domains [27,28,29,30,40,41,42]. The same mechanism could be applied to the PNIPAAm/PDMAAm co-crosslinking gels. Namely, PDMAAm domains functioned as water pathways to induce much more rapid shrinking of the co-crosslinking gel than that of the copolymerization gel, in which hydrophilic monomer units randomly distributed and domain structure was not formed.

## 3. Conclusions

We evaluated the effect of monomer sequence along the network chain of polymer gels on the swelling behavior by utilizing two gel synthetic methods: copolymerization and co-crosslinking. We clearly demonstrated that the copolymerization gels and the co-crosslinking gels showed different swelling behavior even with the same monomer composition in a series of monomer combinations including hydrophilic/hydrophobic, thermoresponsive/hydrophobic, and thermoresponsive/hydrophilic acrylamide derivatives. Importantly, the copolymerization method afforded random monomer distribution to change the affinity of each network chain to water depending on monomer composition. On the other hand, the co-crosslinking method produced a blocky sequence derived from two kinds of prepolymers, which separately and independently contributed to the swelling properties such as the degree of swelling and transition temperature. Thus, we clarified the effect of monomer sequence along the network chain on the properties of polymer gels. These fundamental results are important for the design of polymer gel materials such as stimuli-responsive materials and biomedical devices in combination with plural monomers.

## 4. Materials and Methods

### 4.1. Materials

DMAAm was kindly provided by Kohjin Co., Ltd. (Tokyo, Japan), and it was purified by distillation before use. NBAAm was prepared by the reaction of acryloyl chloride with *n*-butylamine, and purified by distillation before use. NIPAAm (Wako, Osaka, Japan, 98%) was purified by recrystallization from toluene/*n*-hexane. NHSA was prepared as reported in literature [43]. BIS (Wako, 99%), EDA (Wako, 98%), azobisisobutyronitrile (AIBN; Wako, 98%), 1,2,3,4-tetrahydronaphthalene (tetralin; Aldrich, St. Louis, MI, USA, 99%), 1,4-dioxane (Wako, 99%), DMF (Wako, 99%), and methanol (Wako) were used as received.

### 4.2. Copolymerization Gel Synthesis

A typical example of gel synthesis by copolymerization is given below. DMAAm (0.243 g), NBAAm (0.312 g), and BIS (52.5 mg) were all dissolved into methanol (7.0 mL). After addition of AIBN (63.9 mg), the solution was transferred into a test tube containing glass capillaries (volume: 40 μL, internal diameter: 1.3 mm) and bubbled with nitrogen for 10 min. The test tube was immersed in the water bath controlled at 55 °C and kept for 24 h for the completion of the gelation. Under these conditions, almost all of the monomers were consumed to become the gel, and the network polymer composition was considered to be equal to the monomer concentration ratio. Afterwards, the cylindrical gels were taken out from the capillaries and washed with distilled water by means of immersion for several days.

### 4.3. Co-crosslinking Gel Synthesis

A typical example of gel synthesis by co-crosslinking is given below. DMAAm (1.95 mL), NHSA (169 mg), tetralin (0.50 mL), AIBN (32.8 mg), and 1,4-dioxane (7.5 mL) were added to a 50 mL round-bottomed flask equipped with a three-way stopcock and bubbled with nitrogen for 10 min. The flask was then placed in an oil bath kept at 60 °C for 70 min. The reaction was quenched by cooling down the reaction solution to −78 °C. Monomer conversion was determined from the concentration of residual monomer measured by ^1^H NMR with tetralin as an internal standard. Then, the reaction mixture was poured into diethyl ether to obtain the purified P(DMAAm/NHSA) prepolymers. The monomer composition of the polymers was determined by ^1^H NMR analysis. PNBAAm and PNIPAAm prepolymers were also prepared by the same procedures.

The obtained PDMAAm prepolymer [NHSA content: 7.7 mol% (calculated by ^1^H NMR)] (79.3 mg) and the PNBAAm prepolymer [NHSA content: 6.7 mol%] (95.4 mg) were dissolved into DMF (0.50 mL). To this polymer solution, 0.5 mL of EDA/DMF solution (amino groups were set to be equimolar amounts of NHSA units in the prepolymers) was added, and glass capillaries (volume: 40 μL, internal diameter: 1.3 mm) were added to the reaction vessel. The mixture was kept for 24 h to complete the crosslinking reaction. Then, the cylindrical gels were taken out from the capillaries and washed with distilled water by means of immersion for several days to remove the resultant *N*-hydroxysuccinimide, which was produced by the reaction with activated ester and diamine crosslinker.

### 4.4. Characterization

Number-average molecular weight (*M*_n_) and polydispersity index (*M*_w_/*M*_n_) of polymers were determined by size-exclusion chromatography (SEC) in DMF containing 10 mM LiBr at 40 °C using three polystyrene gel columns (PLgel 5 μm MIXED-C, PLgel 3 μm MIXED-E and Shodex KF-805L) that were connected to a Shimadzu LC-10AD precision pump (Kyoto, Japan) and a Shimadzu RID-10A refractive index detector (Kyoto, Japan). The columns were calibrated against standard poly(methyl methacrylate) samples (Agilent, Santa Clara, California, USA). ^1^H NMR spectra were recorded on a JEOL JNM-LA400 spectrometer (Akishima, Japan), operating at 399.65 MHz. The degree of swelling was determined by measurement of the diameter of the cylindrical gels. The gels were immersed in water at predetermined temperatures, and the equilibrium diameter at given temperature, *d*, was measured using a digital microscope (MOTICAM2000, Shimadzu, Kyoto, Japan). The degree of swelling was calculated by (*d*/*d*_0_)^3^; *d*_0_ is the inner diameter of the capillary used for the gel preparation. The shrinking rate was evaluated by temperature-jump experiment. The cylindrical gels were immersed in water at 5 °C until the gels reached to the equilibrium swollen state. Then, the gels were quickly transferred into the hot water at 50 °C, and the change of the diameter of the gel was observed.

## Figures and Tables

**Table 1:** Prepolymers for co-crosslinking gels prepared by radical copolymerization with NHSA. ^1^

Prepolymer	NHSA Content (%) ^2^	*M*_n_^3^	*M*_w_/*M*_n_^3^
PDMAAm	7.7	43,900	1.78
PNBAAm	6.7	53,900	1.71
PNIPAAm	6.4	76,700	1.50

^1^ The polymerization was conducted with the condition below: [acrylamide derivative] = 1900 mM, [NHSA] = 100 mM, [azobisisobutyronitrile (AIBN)] = 20 mM in 1,4-dioxane at 60 °C. ^2^ Determined by ^1^H NMR analysis. ^3^ Determined by size-exclusion chromatography (SEC) analysis with poly(methyl methacrylate) standard. PDMAAm = poly(*N*-dimethylacrylamide); PNBAAm = poly(*N*-*n*-butylacrylamide); PNIPAAm = poly(*N*-isopropylacrylamide).
